# Assessment of Ventriculoperitoneal Shunt Function Using Ultrasound Characterization of Valve Interface Oscillation as a Proxy

**DOI:** 10.7759/cureus.2205

**Published:** 2018-02-19

**Authors:** April Aralar, Matthew Bird, Robert Graham, Beomseo Koo, Parag Chitnis, Siddhartha Sikdar, Mahesh Shenai

**Affiliations:** 1 Bioengineering, University of California, San Diego Test; 2 Bioengineering, George Mason University; 3 Biomedical Engineering, University of Michigan; 4 Neurosurgery, Inova Fairfax

**Keywords:** hydrocephalus, noninvasive, shunt failure detection, ultrasound

## Abstract

Objective

Ventricular shunts are a mainstay of hydrocephalus treatment, but the detection of its clinical failure often relies on circumstantial evidence. A direct, non-interventional method for reliably evaluating cerebrospinal fluid (CSF) function does not exist due to the difficulty of measuring in vivo flow characteristics. The objective of this study is to apply a novel method of ultrasound monitoring to characterize the oscillation observed during pulsatile CSF flow and failure states in an in vitro and cadaveric model.

Method

In this proof-of-concept report, ultrasound is utilized to noninvasively monitor the shunt valve and characterize its mechanical response to different flow conditions. In vitro and in situ testing was carried out by running deionized water through a ventriculoperitoneal shunt (VPS) system using a pulsatile flow generator to replicate the flow rates expected in vivo. Different flow conditions were then tested: no flow, normal flow, proximal obstruction, and distal obstruction. Ultrasound data taken from the pressure relief valve were analyzed to determine differences in the displacement of valve components over time between flow states.

Results

Displacement patterns of the four different flow conditions were determined by directly tracking the changes from the M-mode plots. Each pattern was found to be distinct and repeatable with statistically significant results found when comparing the normal flow condition to distal and proximal obstruction cases.

Conclusions

Each of the flow conditions was found to have a distinct displacement profile, demonstrating that ultrasound imaging of the shunt valve can be used to accurately differentiate between flow and failure conditions. Ultrasound monitoring may be a promising adjunct approach in determining the need for surgical shunt exploration.

## Introduction

Ventriculoperitoneal (VPS) shunts have become indispensable in the treatment of many neurosurgical conditions. However, the sporadic malfunction of shunts provides a significant source of clinical uncertainty. Presenting clinical symptoms of shunt malfunction are often indistinguishable from common maladies with non-specific symptoms. Changes in ventricular size, as detected by computed tomography (CT) or magnetic resonance (MR) imaging, may be absent in approximately 10-30% of shunt failures [1-2]. Clinically, practitioners may utilize the shunt “tap” method to invasively assess the shunt system – but this test has a low negative predictive value [3]. A positive nuclear study may be able to determine no or low flow states, but it would fail in specifically differentiating between proximal and distal occlusions.

Determining valve patency is technically difficult primarily because the flow of cerebrospinal fluid (CSF) is slow, with an equilibrium flow rate of approximately 500 ml/day (0.4 ml/min) on average, and intermittent, depending upon the driving pulsatile intracranial pressure (ICP), which ranges from 5-15 mmHg in a supine adult [4]. Active flow in a perfectly functional shunt may not occur when the ICP is below the valve pressure threshold and the instantaneous flow through the shunt would vary during the cardiac cycle. Therefore, the clinician is forced to deduce the patency of a shunt by interpreting multiple objective and subjective data points. The gold standard for the determination of shunt malfunction remains surgical exploration, which has high associated costs, a risk of infection, and may be unnecessary if the shunt is found to be functional following the removal [5].

A number of non-invasive techniques have been proposed to evaluate shunt function. ShuntCheck (Neuro Diagnostic Devices, Inc, Trevose, PA) is a commercially available device that detects a temperature change of the skin overlying the shunt, downstream from a region cooled by a cold source. Flow is detected by reading the change in the surface temperature, assuming a direct relationship between fluid flow and temperature transfer. However, a clinical study by Madsen et al. [6] failed to show reliability to predict the need for surgery. Another method utilizes the phase shift in otoacoustic emissions observed after tilt-table postural changes [7] and has been described as an indirect, albeit potentially accurate, proxy of shunt function. However, the method has been tested only on the idiopathic normal pressure hydrocephalus (NPH) patient population, and its utility has yet to be determined in the context of diverse pathophysiology.

The use of ultrasound imaging in identifying shunt function was proposed in the early 1980s [8], but the modality has failed to gain clinical adoption. In more contemporary studies, Hartman et al. [9] demonstrated ex vivo that contrast-enhanced ultrasound could be useful in detecting physiologic flow, or lack thereof, in a malfunction state. The feasibility of Doppler measurements has also been described [10], which uses microbubble contrast to enhance the measurement. However, the use of ultrasound to assess shunt function has relied primarily on an assessment of flow, which is intermittent and of low magnitude, even in normally functioning shunts. Furthermore, this technique requires the injection of contrast agents into the CSF or the shunt cavity.

In this paper, we investigate the feasibility of using an alternative ultrasonic approach to assessing shunt function by characterizing the movement of the silastic shunt valve interface in response to pulsatile CSF flow [11] using a clinical ultrasound system. We hypothesize that a properly functioning shunt valve would predictably displace in response to a pulsatile pressure change in the shunt system. In this work, we investigated whether normal flow, no flow, and both proximal and distal obstructions can be differentiated based on ultrasound measurements of the valve displacement in in vitro and cadaveric models. Understanding the ability of ultrasound to detect these flow states could lead to a direct but non-invasive method of shunt function evaluation.

## Materials and methods

In this work, we adapted and optimized our ultrasound-based method that was used for assessing shunt function in vitro [12] for demonstrating the feasibility of this approach in a cadaveric model in situ. In the following sections, we describe the updated materials and methods that were used in cadaver testing and provide a comparison between two methods of determining valve displacement from ultrasound data. In order to ensure experimental results that were relevant for future translation of these techniques in clinical applications, physiologically realistic CSF flow was generated using a custom-built pulsatile fluid flow generator. The shunt models tested were the Medtronic CSF-flow control valve (Medtronic, Minneapolis MN) (in vitro experiments) and the Medtronic Delta® valve (cadaveric study).

Pulsatile flow generator

Pulsatile fluid flow was generated by using an intravenous (IV) drip system connected to a circular motor-driven rotational arm, controlled for a frequency to approximate the heart rate (1.2 Hz). The average hydrostatic pressure was adjusted by changing the difference in height between the center of the rotational arm and the level of the valve, whereas the pulse pressure was adjusted by the radius of the rotational arm, to range between 10 mmH_2_O to 200 mmH_2_O, at the extremes of the sinusoidal waveform. Physiological ICP range is dependent on a number of factors, such as age, posture, and other clinical conditions [13]. Therefore, a wide pressure range was investigated to account for the variation between individuals.

Ultrasonic measurement of valve displacement

A SonixTOUCH ultrasound system (BK Ultrasound, Peabody, MA) with an L40-8/12 high frequency (bandwidth of 8-40 MHz) linear array transducer was used. The transducer was selected due to the smaller footprint and higher spatial resolution as compared to the conventionally used linear array transducers. The transducer was positioned above the shunt valve and reservoir interfaces longitudinally in order to observe the valve location and then oriented transversely over the valve to observe the interfaces associated with the valve cross-section. Optimal transducer placement directly over the valve is necessary to achieve high sensitivity to valve displacement. Positive displacement was defined as valve movement towards the transducer, while negative displacement was defined as movement away from the transducer. Slight deviations in the transducer placement would create decreased signal strength due to the changing angle of incidence.

In vitro experiments

**Figure 1:**
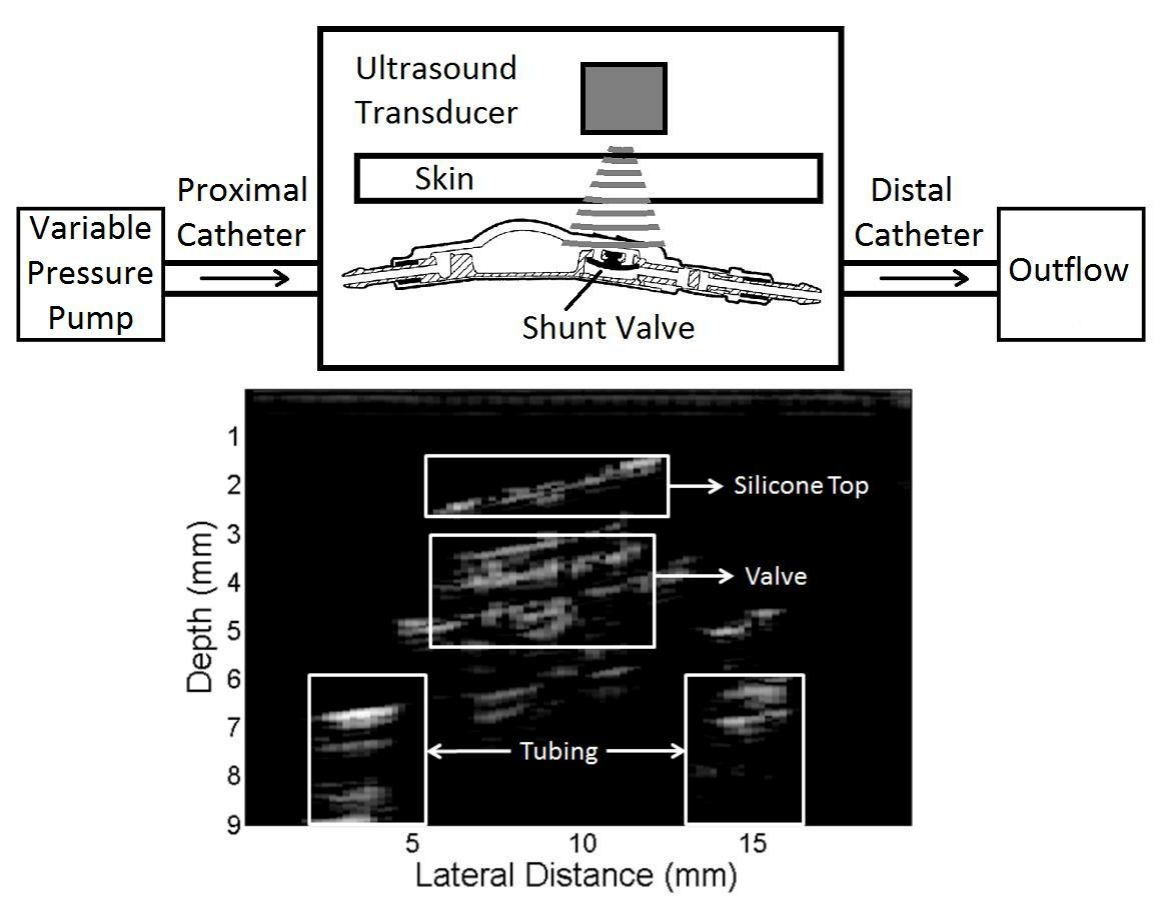
Schematic of the in vitro set up and the corresponding B-mode image Top: Schematic diagram of the in vitro set up which shows fluid flow through the variable pressure pump, into the proximal catheter, then into the reservoir and valve, and drains out through the distal catheter. The artificial skin was placed over the reservoir and valve, and images were taken with the transducer clamped transversely over the valve. Bottom: B-mode image showing the various layers of the shunt which were resolved using a high-frequency ultrasound transducer. The valve was identified by locating an area of consistent axial movement at the input frequency.

A low pressure rated CSF-Flow Control valve (Medtronic, Minneapolis, MN) was used for a majority of testing. The valve was secured above an acoustically opaque platform to emulate an implanted valve above the skull. Inflow to and outflow from the valve was delivered by standard silastic shunt tubing (Codman Neuro, Raynham, MA). Artificial skin with 2N puncture toughness (SKU 141500) (Syndaver, Tampa, FL) was placed above the shunt to simulate the in vivo environment. All surfaces were covered with ultrasound gel to prevent reverberation by air. The distal catheter drained into a secondary tank. The ultrasound transducer was rigidly positioned over the silicone-membrane valve with use of a C-clamp. Positional data of the silastic valve interface was collected and stored on an external hard drive (Western Digital, Irvine, CA). A schematic of the in vitro set up and the corresponding B-mode image is shown in Figure 1.

Cadaveric preparation

**Figure 2:**
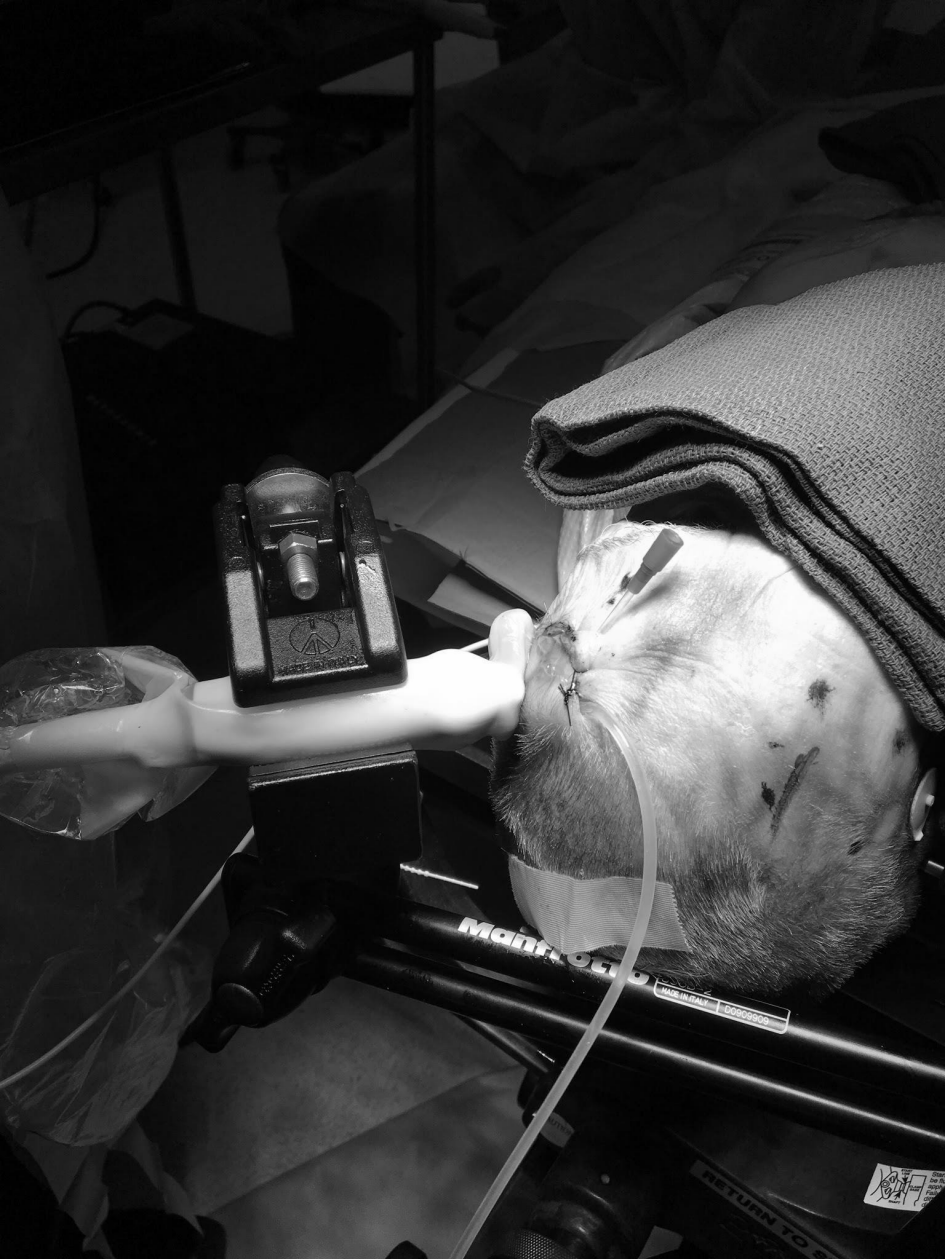
Cadaver set up The transducer is manually positioned over the valve and clamped into place to minimize displacement shifts.

A fresh, frozen cadaver specimen (head, neck, clavicle) was obtained and brought to room temperature. The head was secured using Mayfield three-point fixation and secured to a standard operating room bed. A curvilinear incision was made with a scalpel over the left frontal region, and the soft tissue was bluntly dissected posterior to the incision to create a pocket for the shunt valve. A shunt tunneling device was used to connect the cranial incision with an exit point below the clavicle of the specimen. A silk tie was then pulled from the exit point to the cranial incision and tied to the distal end of silastic tubing, itself connected to a pre-assembled Delta “low pressure” flow control valve (Medtronic, Minneapolis, MN). The shunt valve was then situated in the pocket. The proximal end of the valve was connected to standard silastic tubing which exited from the incision and connected to the pulsatile flow generator. The incision was closed with a running 3-0 silk suture. An additional catheter was placed in the subcutaneous space to introduce ultrasound gel into the testbed and to displace any air that would interfere with ultrasound imaging. The cadaver set up is shown in Figure 2 with the placement of the transducer in relation to the implanted valve and its catheters.      

 

Flow state simulation

Several states of shunt function were examined: A) no flow, B) proximal obstruction, C) normal flow, and D) distal obstruction. All obstructions (B and D) were simulated by the use of A-clamps to produce a discrete closure on either the proximal or distal catheter. The obstructions were confirmed by observing a cessation of fluid flow from the distal catheter after a transition period of 10 minutes. Transient flow states, where recording occurred during the induced obstruction, were used to confirm how quickly a steady state was achieved. Transducer alignment with the valve was confirmed by manually creating flow perturbations by compressing the distal catheter. The perturbations caused a backflow that closed and reopened the valve by siphoning deionized water, which created a reproducible movement at the valve interface.

Data analysis

**Figure 3:**
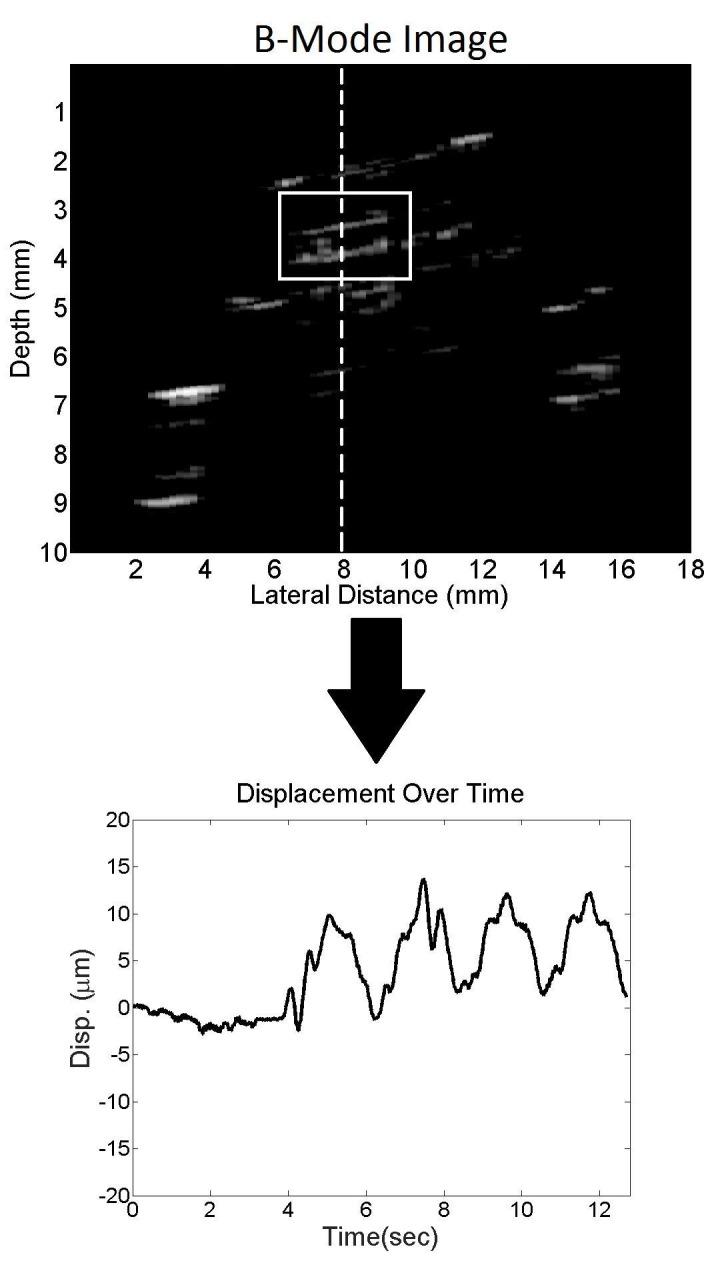
B-mode image Top: A B-mode image of shunt cross-section. The white box indicates the valve region of interest; the white dashed line denotes the scanline analyzed. Bottom: Displacement (disp) of the shunt valve interface over time derived from B-mode image sequence.

Raw radio frequency (RF) ultrasound data collected from the SonixTOUCH were analyzed offline using MATLAB (MathWorks Inc., Natick, MA). The location of the valve interface was visualized on the reconstructed grayscale (B-mode) images and a region of interest was manually marked. An example B-mode image showing the valve area as outlined by the white rectangle and the vertical scan line selected is shown with the corresponding displacement calculation in Figure 3. Small movements of the valve interface introduced changes in the phase of the received RF ultrasound echoes. The time-varying valve displacements were calculated by interpolating the M-mode image by a factor of 1,000 and tracking the brightest pixel within the region of interest. Displacement plots were filtered using a 15 point moving average filter. Valve displacements were also estimated from the phase differences between consecutive RF echoes as a method of cross-validation to ensure statistically significant results. We utilized the autocorrelation method [14] to estimate phase differences from the demodulated RF data. The cumulative displacement of the valve interface over time was generated by accumulating the instantaneous displacement between consecutive ultrasound echoes at the selected depth. Linear detrending of the signal was performed to remove baseline drift caused by the accumulation of noisy estimates.

## Results

In vitro testing

Testing of the transient flow states confirmed that steady-state flow conditions were attained within the shunt system immediately after each obstruction was induced. Steady-state was characterized by the lack of visible change in the displacement profile of the valve. All subsequent test results are described for the steady-state condition of each flow type. The “no-flow” and “normal flow” cases were used as control conditions. The “no-flow” case allowed for a baseline measurement of the various interfaces in the valve. Under this condition, since no valve displacement was observed, a manual compression of the distal catheter was performed to force a movement of the valve interface and confirm that the transducer was properly aligned with the valve. These introduced perturbations were well-visualized, and the displacements generally ranged between (30 - 50 μm).

By contrast, the “normal flow” case demonstrated periodic and robust pulsatile displacement with peak-peak amplitudes of 11.771 ± 16.451 μm. The periodicity of the waveforms exactly correlates with the set periodicity of the pulse generator. By comparing the displacement patterns for each induced flow state, we have determined that each state can be differentiated in a reproducible manner.

**Figure 4:**
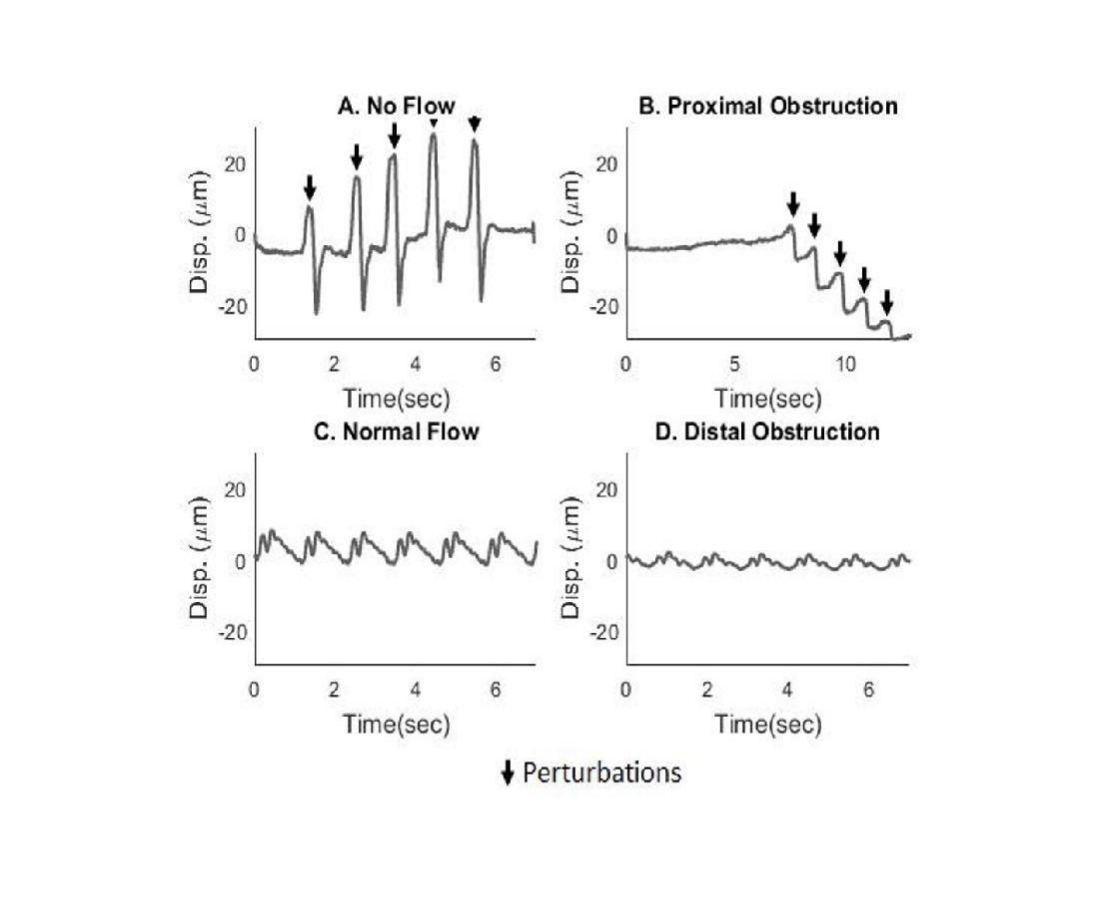
Displacement profiles of four flow states were compared in vitro with the system initially beginning at rest with no-flow through the shunt, in the no-flow and proximal obstruction cases (A) The no-flow case shows no overall displacement (disp) changes. Five manual perturbations were induced, which are indicated by the arrows, and the baseline remains around 0. (B) The proximal obstruction case shows a steady baseline with no periodic displacement as in the no-flow case.  Five manual perturbations were induced, which are indicated by the arrows. In this case, the perturbations cause the baseline to decrease. (C) The normal flow case shows periodic behavior at an amplitude of about 10 μm. (D) The distal obstruction flow case also shows a periodic but decreased amplitude from the normal flow condition of about 5 μm.

In Figure 4, the in vitro no-flow (A) and proximal obstruction (B) cases are shown with perturbations to ensure transducer placement over the valve. In both cases, there is no periodic, pulsatile pattern, indicating that no fluid is entering the system. In the case of a proximal obstruction, there is a drift in displacement away from the baseline. In the normal flow case (C), periodic, pulsatile flow can be seen with an amplitude about 10 μm. The distal obstruction (D) has a much smaller amplitude than the normal flow case of about 5 μm.

In the “proximal obstruction” tracings, the pulsatility of the valve motion was not visible. The displacement returned a flat signal similar to that of the no-flow case. The measured mean displacement amplitude was found to be 3.773 ± 3.431 μm. Induced perturbations were also well-visualized, but a downward trend in the displacement was observed, showing that water was siphoned out of the reservoir as the perturbations were introduced.

**Figure 5:**
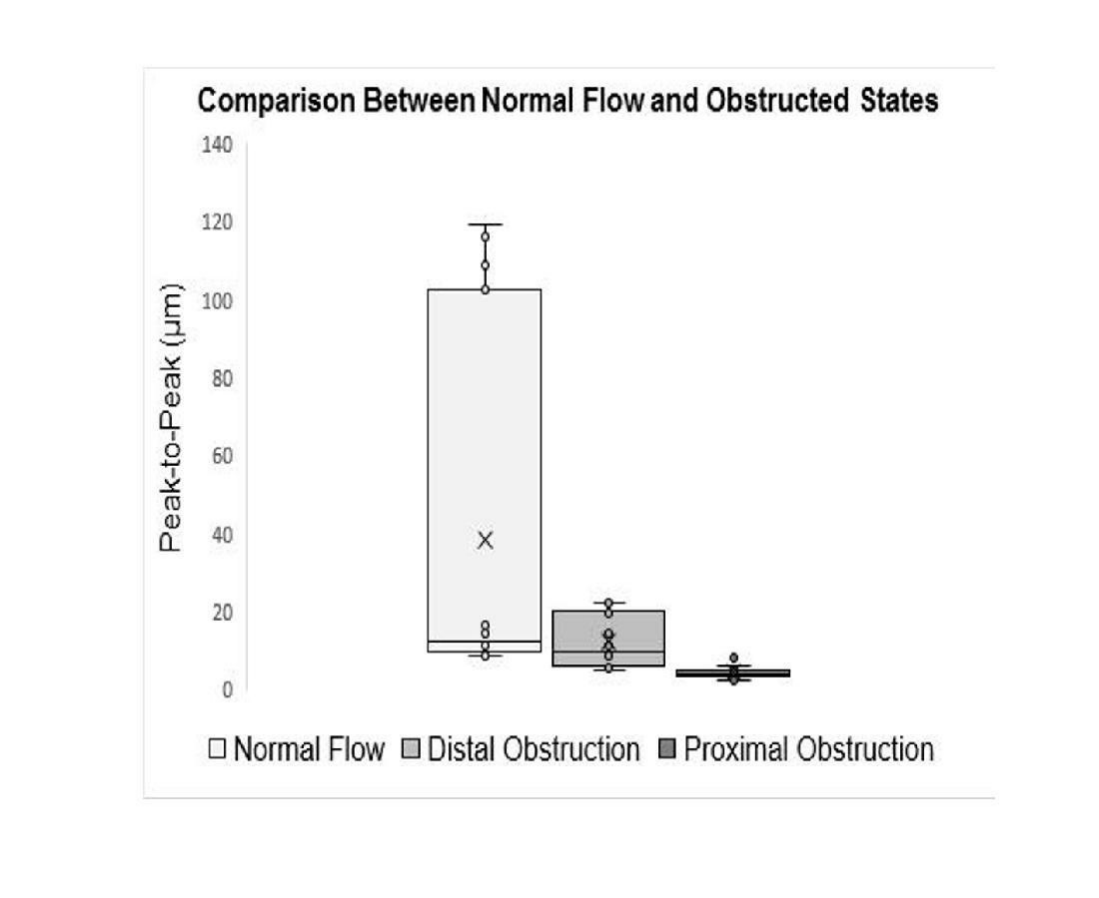
A numerical comparison of the maximum amplitudes of the normal flow, the distal obstruction, and proximal obstruction displacement profile for 15 independent trials Paired, two-tailed t-tests gave a statistically significant p-value < .05 when the normal and distal obstruction conditions, as well as the normal and proximal obstruction conditions, were tested.

In the “distal obstruction” tracings, periodic behavior was still observed similar to the normal flow case, with a mean displacement amplitude of 11.150 ± 14.544 μm. It was found that inducing a distal obstruction into the system would cause the amplitude of the displacement signal to decrease. The difference in amplitude between the normal flow and each of the obstruction conditions were found to be statistically significant. A p value < .05 was found when comparing the normal flow cases against the distal obstruction cases and the normal flow cases against the proximal obstruction cases with paired, two-tailed t-tests. The differentiation between these cases for 15 independent trials is shown in Figure 5, and a clear distinction between each flow case can be visualized. Additionally, although the amplitude values are not equal between both direct M-mode tracking and the conventional autocorrelation technique, there are still statistically significant results when comparing the normal flow cases with the distal and proximal obstruction cases. The absolute displacement values are irrelevant due to the displacement sensitivity to transducer placement, with statistical significance when comparing flow types being the most important consideration. However, in order to make these comparisons, the transducer needs to remain stationary in order to calculate the relative difference between the cases. The peak-to-peak mean and standard deviation and root mean square (RMS) mean and standard deviation values are listed in Tables 1 and 2.

**Table 1:** Average Peak-to-Peak and RMS Displacement Amplitudes of Each of the Three Flow Conditions are Listed for 15 Independent Trials When Displacement is Calculated by Tracking Displacements Directly From the M-Mode

M-mode Tracking	Peak-to-peak amplitude (μm) Mean ± standard deviation	Root-mean square (RMS) amplitude (μm) Mean ± standard deviation
Normal	38.340 ± 45.975	10.468 ± 13.971
Proximal	4.232 ± 1.503	0.874 ± 0.242
Distal	12.397 ± 6.750	2.902 ± 1.887

**Table 2:** Average Peak-to-Peak and RMS Displacement Amplitudes of Each of the Three Flow Conditions are Listed for 15 Independent Trials When Displacement is Calculated by the Conventional Autocorrelation Method to Estimate Phase Changes

Autocorrelation Calculation	Peak-to-peak amplitude (μm) Mean ± standard deviation	Root-mean square (RMS) amplitude (μm) Mean ± standard deviation
Normal	4.424 ± 2.379	1.000 ± 0.547
Proximal	0.284 ± 0.063	0.049 ± 0.010
Distal	2.596 ± 1.372	0.586 ± 0.293

In situ testing

**Figure 6:**
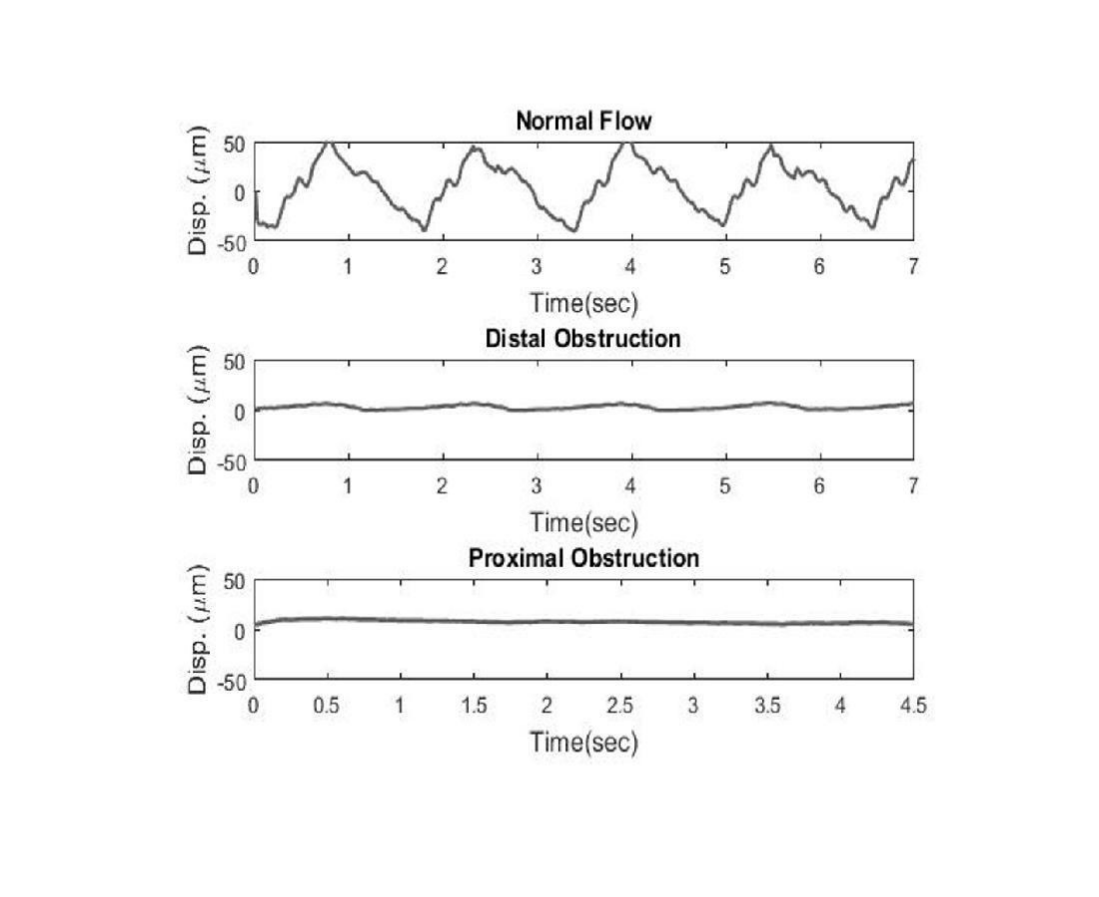
Displacement (disp) profiles of flow validation tests in situ for the three flow conditions of normal flow, distal obstruction, and proximal obstruction. The observed patterns in the displacement profiles in situ return the same patterns found in in vitro testing.

Figure 6 shows a validation of the different in situ flow conditions, where the same flow conditions were imitated within a cadaveric model. As seen in the in vitro testing, the normal flow conditions produced a pulsatile displacement pattern but with a displacement amplitude around 100 μm. The distal obstruction condition also showed a pulsatile displacement pattern but with a decreased amplitude of about 10 μm. Finally, the proximal obstruction condition showed no pulsatile behavior and no displacement pattern, returning only a flat signal.

## Discussion

Ultrasound monitoring was utilized in this study because it is relatively cheap, quick, and easy to implement, portable, and poses minimal risk. Our preliminary results show that ultrasound assessment of valve interface oscillation can provide a specific predictor of shunt function. The induced flow states were readily observed in the displacement profile, where the pulsatility of the silicone membrane valve interface in the normal CSF flow case was seen in B-mode and M-mode ultrasound images, while the no-flow state failed to reflect pulsatility. A cessation of flow was also observed during the proximal obstructions. Endpoint analysis of clinical shunt malfunctions states that proximal obstructions account for 27% of revisions, compared to the 15% associated with distal obstructions [14]. Because our method can readily differentiate between normal CSF periodic flow state and proximal obstruction, diagnosis of the most common shunt failure state proves possible by our method.

Distal obstructions continued to show pulsatility; however, the waveforms reflected a decreased amplitude from the normal flow condition. This phenomenon likely results from the continuity of the valve with the pressure wave that is created by the generator, allowing incremental flow to be introduced into the valve up until the absolute volume limit of the valve. Since the amplitude of both the normal and distal obstruction waveforms are dependent on transducer placement, there is no absolute threshold that distinguishes the distal obstruction condition. For these cases, a transient test between normal and distal obstruction is needed, and numerical comparison of the amplitude of the displacement can be used to identify the presence of a distal obstruction. Since a steady-state is reached immediately after an obstruction is induced, only a few seconds worth of data collection would be necessary to obtain the displacement pattern. The presence of a decreased displacement amplitude would indicate that there is no obstruction within the system. If the system is perturbed and no change in amplitude is observed, then this would indicate that there is already a distal obstruction present. These distal perturbations can be produced in vivo by manually pressing on the catheter over the clavicle and can, therefore, be used as a tool to create the conditions required for numerical analysis of both flow states.

Future work will include the development of methods that will ensure that the placement of the transducer with respect to the VPS valve is optimal and consistent. This is being implemented by the use of a three dimensional (3D) printed cuff in the shape of the valve, which will ensure that the transducer is placed directly over the valve each time, through a hole shaped exactly to each shunt model and for the transducer being used. The transducer placement cuff will allow simple, low-cost implementation of our ultrasound monitoring technique to a clinical setting.

Limitations

Since this method relies on movement of the valve mechanisms, it can be extended to any VPS model that employs a moving part, which encompasses most models of VP shunts. Currently, there are four basic valve designs: slit, miter, diaphragm, and spring-loaded ball in cone valves [15]. The valves tested were all of the diaphragm design, which consists of a flexible silicone membrane that responds to pressure differences in the valve and only allows fluid to flow when a pressure differential is present. The spring-loaded ball in cone valve design, used in the Strata® valve (Medtronic, Minneapolis, MN) and the Polaris® valve (Sophysa USA, Inc., Crown Point, IN), consists of a plastic ball which pushes against a metallic coil or flat spring, allowing fluid to flow if the pressure gradient is reached. Since the metallic coil sits under the valve, ultrasound can still be used to visualize fluid flow through the valve.

The slit valve design, used in the Spetzler™ lumbar peritoneal shunt system (Integra NeuroSciences, Inc., Plainsboro, NJ), consists of a cut in the wall of the distal catheter which opens if there is a sufficient pressure differential. The miter valve design, such as that used in the Mishler™ dual chamber valve and the UltraVS™ cylindrical valve system (Integra NeuroSciences, Inc., Plainsboro, NJ), consists of a small outlet in a vertical reservoir chamber divider which allows CSF to flow if the pressure control setting is exceeded. Since there are no moving parts in these models, our method would not be usable; however, these are older valve models which are used less frequently than the diaphragm and ball in cone models. Further testing would need to be completed to determine if this method is extendable to different valve brands.

Another current limitation of the technology is the variability introduced because of the placement of the ultrasound transducer relative to the valve interface. We are developing customized transducers that can be more reproducibly placed over the shunt which will decrease variability. Additionally, shunt malfunction can be caused by a number of other factors, such as partial obstructions, infection, catheter migration, underdrainage, etc. [16]. Finally, physiological CSF flow is intermittent, which would result in displacement flow patterns that differ from the expected cases, complicating the determination of the presence of an obstruction. Continued testing is needed to simulate other causes of shunt malfunction and the intermittent nature of CSF flow within shunts to determine if this method is successful in identifying failure states in these instances.

## Conclusions

This study demonstrates that (1) ultrasound-based measurement of the displacement of the VPS valve membrane can be used to detect a deviation from normal flow through the valve, and (2) the displacement characteristics are different between proximal and distal obstructions. This approach could potentially lead to a noninvasive method for examining shunt function in vivo.
